# Si-Miao-Yong-An Decoction Protects Against Cardiac Hypertrophy and Dysfunction by Inhibiting Platelet Aggregation and Activation

**DOI:** 10.3389/fphar.2019.00990

**Published:** 2019-09-18

**Authors:** Congping Su, Qing Wang, Huimin Zhang, Wenchao Jiao, Hui Luo, Lin Li, Xiangyang Chen, Bin Liu, Xue Yu, Sen Li, Wei Wang, Shuzhen Guo

**Affiliations:** ^1^School of Traditional Chinese Medicine, Beijing University of Chinese Medicine, Beijing, China; ^2^School of Chinese Materia Medica, Beijing University of Chinese Medicine, Beijing, China; ^3^School of Life Sciences, Beijing University of Chinese Medicine, Beijing, China

**Keywords:** Si-Miao-Yong-An decoction, cardiac hypertrophy, heart failure, platelet activation, transverse aortic constriction

## Abstract

**Objective:** The aim of this study was to determine whether Si-Miao-Yong-An decoction (SMYAD) could ameliorate pressure overload-induced heart hypertrophy and its mechanisms.

**Methods:** C57BL/6 mice were subjected to either sham or transverse aortic constriction (TAC) surgery to induce heart hypertrophy. SMYAD (14.85 g/kg/day, ig) or captopril (16.5 mg/kg/day, ig) was administered to the mice for 4 weeks. Cardiac function was evaluated based on echocardiography. Heart hypertrophy was detected using hematoxylin and eosin or wheat germ agglutinin staining. Protein expression of CD41, CD61, and P-selectin were measured with Western blot and immunohistochemistry. The expression levels of atrial natriuretic peptide, brain natriuretic peptide, β-myosin heavy chain, β-thromboglobulin, and von Willebrand factor were evaluated by quantitative polymerase chain reaction.

**Results:** Four weeks after TAC, mice developed exaggerated cardiac hypertrophy and demonstrated a strong decrease in left ventricular ejection fraction compared with sham (29.9 ± 9.3% versus 66.0 ± 9.9%; *P* < 0.001). Conversely, SMYAD improved cardiac dysfunction with preserved left ventricular ejection fraction (66.5 ± 17.2%; *P* < 0.001). Shortening fraction was increased by SMYAD, while the left ventricular internal diameter and left ventricular volume were decreased in SMYAD group. SMYAD treatment significantly attenuated cardiac hypertrophy as reflected by the inhibition of atrial natriuretic peptide, brain natriuretic peptide, β-myosin heavy chain mRNA expression, and by the decreasing of cardiac myocyte cross-sectional area. Furthermore, Western blot and immunohistochemistry indicated that the protein expression of platelet aggregation markers (CD41 and CD61) and platelet activation marker (P-selectin) were significantly higher in model mice compared with control. These pathological alterations in TAC-induced mice were significantly ameliorated or blocked by SMYAD administration.

**Conclusions:** Our results suggested that SMYAD exerted its effect by inhibiting platelet aggregation and activation as revealed by CD41/CD61/P-selectin downregulation. Inhibition the activation of the platelets might contribute to the therapeutic effect of SMYAD in failing heart.

## Introduction

Cardiac hypertrophy is a common pathophysiological component of cardiac remodeling in many kinds of cardiovascular diseases, such as valvular heart disease, hypertension, and hypertrophic cardiomyopathy (Pillai et al., 2015). This process is usually considered to be a compensatory response of the heart to an increased hemodynamic load, which could cause contractile depression, ventricular dilatation, interstitial cardiac fibrosis, and eventually heart failure (HF) (Ooi et al., 2015). Studies have shown that platelet activation played a critical role in several cardiovascular diseases, such as HF (Schafer et al., 2003), coronary artery disease (George et al., 2016), and atrial fibrillation (Lim et al., 2014). Platelet activation is followed by platelet adhesion that is characterized by expression of variety of glycoproteins on platelet surface. In platelet-related thrombogenesis, platelets adhere to injured endothelium undergoing a conformational transition followed by activation and degranulation. This activation results in the combination of fibrinogen with the platelet surface receptors and consequently leading to thrombus formation (Santhakumar et al., 2015), thereby leading to coronary artery disease and HF.

Si-Miao-Yong-An decoction (SMYAD) is a traditional Chinese medicine formula, which consists of *Lonicerae Japonicae Flos* (Jinyinhua), *Scrophulariae Radix* (Xuanshen), *Angelica Sinensis Radix* (Danggui), and *Glycyrrhizae Radix et Rhizoma* (Gancao). SMYAD was reported to reduce the atherosclerosis plaque area, promote the recruitment of vasa vasorum pericytes, and stabilize atherosclerosis vulnerable plaques in ApoE^-/-^ mice (Qi et al., 2019). SMYAD has also been verified to ameliorate the stability of atherosclerotic plaque by lowering blood lipid in rabbit model (Peng et al., 2012). The formula was also reported to have anti-inflammatory and anti-oxidation properties (Wang and Tian, 2014). We previously demonstrated that SMYAD promoted isoprenaline-induced HF through antioxidant effects (Ren et al., 2019). However, the role of SMYAD in pressure overload-induced cardiac hypertrophy has yet not been explored.

In this study, we found that SMYAD attenuated pressure overload-induced cardiac dysfunction through inhibiting platelet aggregation and activation, suggesting SMYAD as a promising therapeutic agent for adverse cardiac remodeling.

## Materials and Methods

### Animals

All the animal experiments were performed in accordance with the “Guide for the Care and Use of Laboratory Animals” by the National Institutes of Health. This study was approved by the Animal Research Ethics Committee of Beijing University of Chinese Medicine (BUCM-4-2018090701-3019). All studies were conducted in line with the approval of the Animal Care Committee of Beijing University of Chinese Medicine. Male C57BL/6 mice, weighing 20–22 g, were obtained from Vital River (Beijing, China, License number: SCXK 2016-0006) and maintained on a 12:12-hour light–dark cycle with free *ad libitum* access to food and water for a 1-week acclimatization period.

### Sample Preparation and Constituents Identification of Si-Miao-Yong-An Decoction

SMYAD was provided by School of Chinese Materia Medica, Beijing University of Chinese Medicine. *Lonicerae Japonicae Flos*, *Scrophulariae Radix*, *Angelica Sinensis Radix*, and *Glycyrrhizae Radix et Rhizoma* were obtained from Anguo Wanlian Chinese Medicine Yinpian Co. Ltd (Hebei, China) and identified by Professor Yuan Zhang. Detailed information of the drug materials and the scan of the vouchers were given in **Supplementary Table 1**. The voucher specimens were deposited in School of Chinese Materia Medica, Beijing University of Chinese Medicine. The SMYAD was prepared with *Lonicerae Japonicae Flos*, *Scrophulariae Radix*, *Angelica Sinensis Radix*, and *Glycyrrhizae Radix et Rhizoma* at a weight ratio of 3:3:2:1 according to the ancient documents. The herbs were extracted twice by refluxing with 10 times of water (volume/weight) for 2 h each time. Then, the extracted solution was filtered. The filtered extracts were mixed together and then concentrated to the relative density for 1.5 g/ml.

The high-performance liquid chromatography characteristic chromatogram of SMYAD has been previously established and used for its quality control (Li et al., 2018). On the basis of macroporous adsorption resin column chromatography, nuclear magnetic resonance, and mass spectrometry (MS), we have isolated and identified 22 compounds from SMYAD, which were 5(*S*)-5-carboxystrictosidine (1), harpagoside (2), geniposide (3), glycyrrhetinic acid (4), glycyrrhizic acid (5), hyperoside (6), liquiritin (7), isoliquiritoside (8), liquiritigenin (9), isoliquiritigenin (10), luteolin (11), quercetin (12), 2-(3-hydroxy-4-methoxyphenyl)ethyl *O*-α-arabinopyranosyl-(1→6)-*O*-α- rhamnopyranosyl-(1→3)-*O*-β-glucopyranoside (13), angoroside C (14), acteoside (15), cinnamic acid (16), ferulic acid (17), (E)-aldosecologanin (18), protocatechuic acid (19), stigmasterol (20), hentriacontanol (21), and daucosterol (22), and 13 compounds were isolated from SMYAD for the first time (Liu et al., 2018).

To further investigate the pharmacokinetic change of SMYAD, we explored the absorbed constituents of water extract of SMYAD in rat plasma using liquid chromatography–MS-ion trap–time of flight method. As a result, 14 compounds were preliminary identified, of which 10 compounds were prototypes of SMYAD, and four were metabolites (Chi et al., 2016). In addition, seven major active ingredients of SMYAD extract (i.e., harpagide, chlorogenic acid, sweroside, loganin, liquiritin, angoroside C, harpagoside) in rat plasma were detected simultaneously by a sensitive ultra-performance liquid chromatography–tandem mass spectrometry method (Liu et al., 2017a).

### Reagents

Captopril was obtained from Beijing Jingfeng Pharmaceutical Group Co. Ltd. (Drug Production Approval Number H20084569). Pentobarbital sodium was purchased from Sigma (St. Louis, USA).

### Groups and Treatment

Mice were randomly assigned to four groups: sham with vehicle (sham), TAC with vehicle (TAC), TAC with captopril (TAC + captopril), and TAC with SMYAD (TAC + SMYAD). Mice in the latter three groups underwent TAC surgery. On day 3 post-surgery, SMYAD was administered at a dose of 14.85 g/kg/day *via* oral gavage administration for 28 days. Captopril, which was used as a positive control, was administered intragastrically at a dose of 16.5 mg/kg/day for 28 days. The sham and TAC groups were fed intragastrically with equal volumes of double distilled water once daily for 4 weeks.

### Transverse Aortic Constriction

Mice were subjected to TAC-induced pressure overload as previously described (Ren et al., 2018). Briefly, the mice were anesthetized with 0.5% pentobarbital sodium (50 mg/kg), then they were orally intubated and placed on a ventilator to maintain respiration. TAC was created using a 6-0 suture banded between the carotid arteries over a 26-gauge needle. The needle was immediately removed after ligation. Sham group animals underwent the carotid arteries separation procedure but without aortic ligation.

### Echocardiography

Four weeks after the TAC surgery, the cardiac function was assessed by the Vevo2100 imaging system (VisualSonics, Canada). Echocardiography was performed with a 30-MHz linear transducer probe (MS500). Images were captured in M-mode using pulse-wave Doppler and tissue Doppler imaging. Offline image analyses were performed using dedicated Visual Sonics Vevo2100 1.6.0 software.

### Histochemical and Immunohistochemical Analyses

The hearts were fixed in 4% paraformaldehyde, embedded in paraffin, and then stained with hematoxylin and eosin (HE). Pictures were acquired by Aperio VERSA scanning system (Leica Biosystems Richmond, Inc.). For immunohistochemistry, the slides were incubated with primary antibodies against CD41 (Glycoprotein IIb) (1:100, Abcam, ab33661, UK), CD61 (Glycoprotein IIIa) (1:100, Abcam, ab210515, UK), P-selectin (1:100, Abcam, ab54427, UK) overnight at 4°C. After the slides were washed with phosphate-buffered saline, they were incubated with the corresponding secondary antibodies for 1 h at 37°C. Photomicrographs of stained sections were digitalized and analyzed by an automated image analysis system (Image-Pro Plus 6.0 software, Media Cybernetics, Silver Spring, USA).

### Immunofluorescent Staining

Immunofluorescent staining was performed using fluorescein isothiocyanate-conjugated wheat germ agglutinin (WGA) (1:200, Sigma-Aldrich, L4895, USA), and cell nuclei were counterstained with 4’,6-diamidino-2-phenylindole (DAPI) (ZLI-9557, ZSGB-BIO, China). For determination of cardiac myocyte cross-sectional areas, 6-µm thick paraffin-embedded sections were stained for membranes with WGA for 30 min and then sealed with DAPI. Images were taken from areas of transversely cut muscle fibers by microscopy under a 400× field.

### Quantitative Polymerase Chain Reaction

SYBR Green quantitative polymerase chain reaction was performed with Bio-Rad CFX96 Real-Time System. Target-specific primers were designed, and their sequences are listed later (each gene symbol is followed by the forward and reverse primers). Forward and reverse primers, complementary DNA template, and the FastStart universal SYBR Green Master Mix (Roche, USA) were mixed to a final volume of 20 μl. The following programm was used: 95°C for 10 min, followed by 40 cycles of 95°C for 15 s and 60°C for 1 min.

β-actin mouse: F:GGCTGTATTCCCCTCCATCG, R:CCAGTTGGTAACAATGCCATGT;
ANP mouse: F: GCTTCCAGGCCATATTGGAG, R: GGGGGCATGACCTCATCTT;
BNP mouse: F: GAGGTCACTCCTATCCTCTGG, R: GCCATTTCCTCCGACTTTTCTC;
β-*MHC* mouse: F: ACTGTCAACACTAAGAGGGTCA, R: TTGGATGATTTGATCTTCCAGGG;
β-*TG* mouse: F: CTCAGACCTACATCGTCCTGC, R: GTGGCTATCACTTCCACATCAG;
*vWF* mouse: F: GCTGGCATGGAATATAAGGAGTG, R: CCAAGCCTACCTGGGCATT.

### Western Blot Analysis

Total protein from heart tissues was extracted using radioimmunoprecipitation assay lysis buffer, and protein concentrations were evaluated using bicinchoninic acid protein assay kit (Applygen, China). The protein was separated using sodium dodecyl sulfate polyacrylamide gel electrophoresis and then transferred onto polyvinylidene difluoride membranes using a wet transfer apparatus (Bio-Rad, USA). The membranes were blocked in 5% nonfat milk incubated with dilutions of anti-CD41 (1:1,000, Proteintech, 24552-1-AP, China), anti-CD62P (1:1,000, Abcam, ab54427, UK), and anti-CD61 (1:1,000, Abcam, ab210515, UK) antibodies overnight at 4°C. The membranes were then incubated for 1 h at room temperature with secondary antibodies (1:5,000, BioDee Biotechnology, DE0601 and DE0602, China). The membranes were detected using an ECL Western-blotting Detection Reagent kit (catalogue RPN2106, GE Healthcare USA), with the LAS-3000 detection system. Protein levels were analyzed using Image Lab software.

### Statistical Analysis

The statistical analysis was performed using SPSS 22.0 statistical software (IBM, Armonk, NY), and the results were presented as mean ± standard error of the mean. Differences between groups were compared by one-way analysis of variance. *P* < 0.05 was considered statistically significant.

## Results

### Si-Miao-Yong-An Decoction Ameliorated Transverse Aortic Constriction-Induced Cardiac Dysfunction

**Figure 1:**
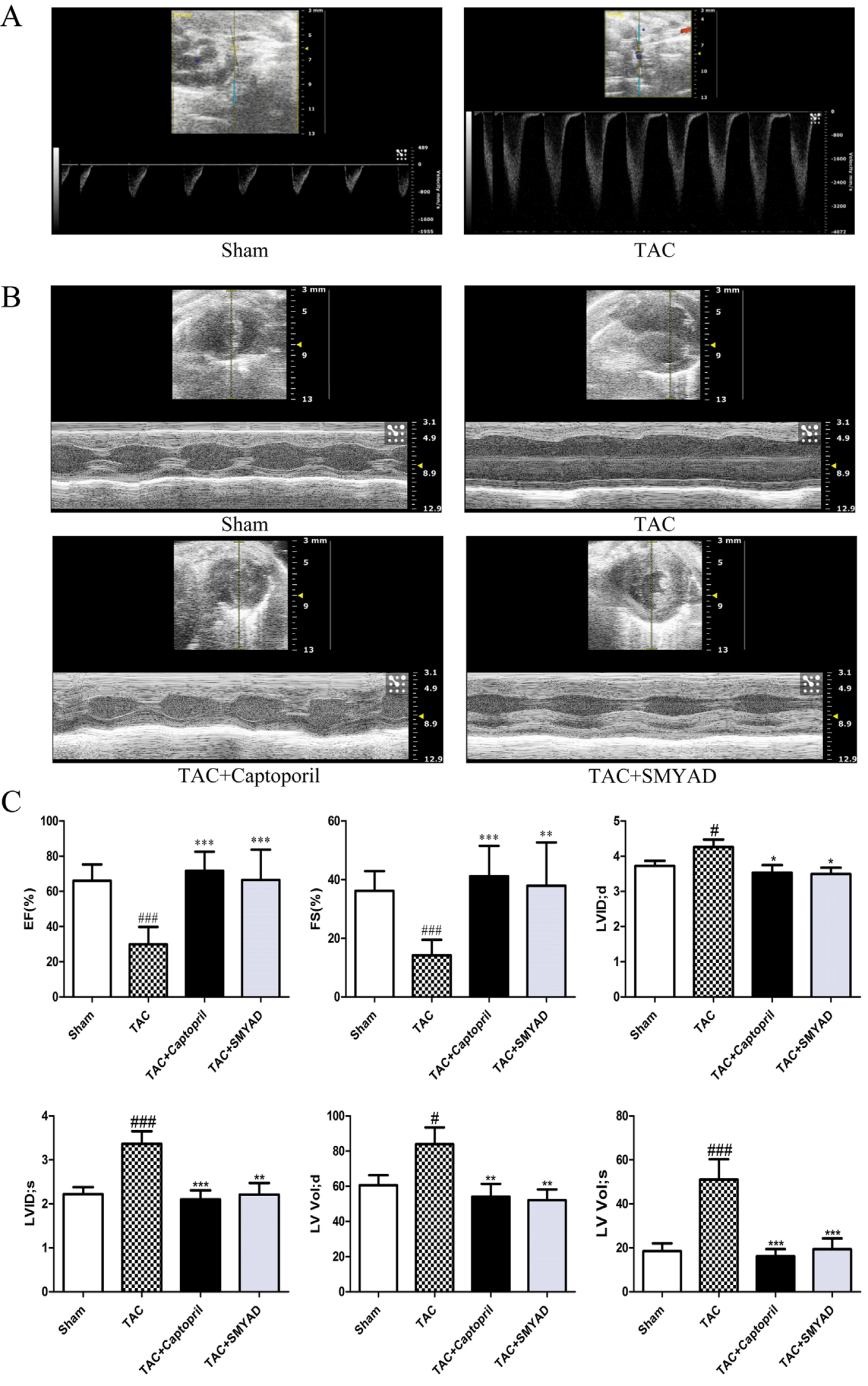
SMYAD improved cardiac dysfunction 4 weeks after TAC. **(A)** Illustrating the transthoracic echo Doppler findings of velocity of blood ﬂow in ascending aorta after TAC procedure. The aortic blood flow rate at the constriction point was greater than 2,400 mm/s in TAC-proceeded mice, while less than 900 mm/s in the sham group. **(B)** Representative M-Mode images. **(C)** Echocardiographic parameter analysis. EF, left ventricular ejection fraction; FS, left ventricular fractional shortening; LVID; d, left ventricular internal diameter at end diastole; LVID; s, left ventricular internal diameter at end systole; LV Vol; d, left ventricular volume at end diastole; LV Vol; s, left ventricular volume at end systole. Sham (n = 12), TAC (n = 8), TAC + captopril (n = 7), TAC + SMYAD (n = 9). ^###^*P* < 0.001 compared with sham, ^#^*P* < 0.05 compared with sham; ***P < 0.001 compared with TAC, **P < 0.01 compared with TAC, *P < 0.05 compared with TAC.

To investigate the effect of SMYAD on the cardiac function, transthoracic echocardiography was performed on the day before the animals were sacrificed (**Figure 1B**). As previously described, the aortic blood flow rate at the constriction point was greater than 2,400 mm/s in TAC-proceeded mice, whereas in the sham group, it was less than 900 mm/s (**Figure 1A**) (Ren et al., 2018). According to echocardiographic evaluation, left ventricular ejection fraction and left ventricular fractional shortening in the TAC group were markedly decreased as compared with those in the sham group, but these two parameters were significantly improved in the TAC + SMYAD group as compared with those in the TAC group (**Figure 1C**). In addition, TAC mice displayed a remarkable increase in left ventricular internal diameter (LVID) compared with the sham-operated mice (*P* < 0.05 and *P* < 0.001 for LVID; d and LVID; s, respectively). In contrast, SMYAD administration effectively reduced the TAC-induced increase in LVID; d and LVID; s by 17.7% (*P* < 0.05) and 34.4% (*P* < 0.01), respectively. Left ventricular volume at end systole (LV Vol; s) was increased after 4 weeks of pressure overload (*P* < 0.001), and SMYAD administration prevented the TAC-induced decrease in LV Vol; s (*P* < 0.001) (**Figure 1C**).

### Si-Miao-Yong-An Decoction Prevented Pressure Overload-Induced Cardiac Hypertrophy

**Figure 2:**
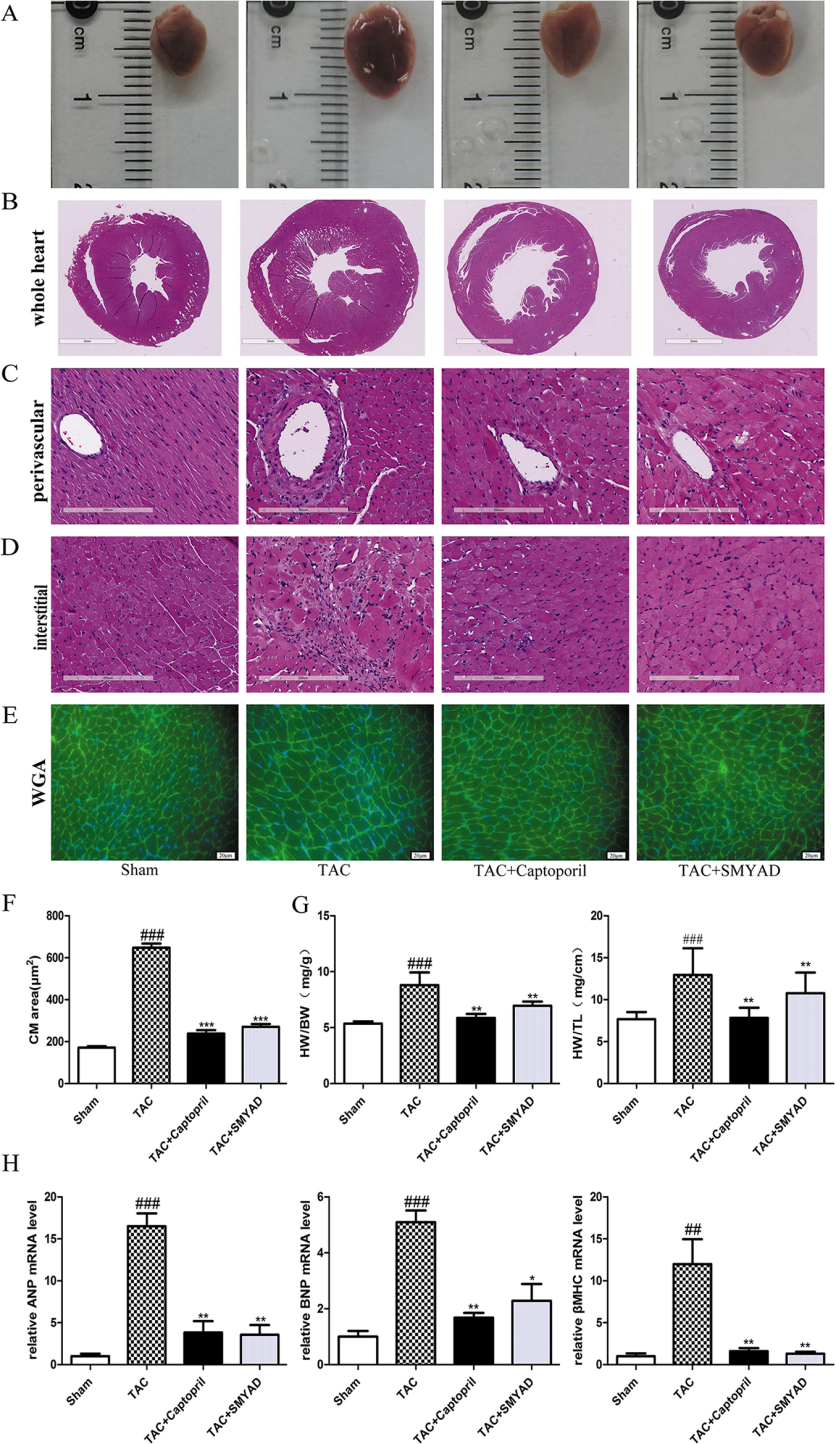
SMYAD reversed cardiac hypertrophy in mice with TAC. **(A)** Representative image of whole heart. **(B**, **C**, and **D)** Representative images of HE staining in whole heart, perivascular, and interstitial. **(E)** WGA staining (green) of histological sections confirmed the inhibitory effect of SMYAD on hypertrophy, and nuclei were counterstained with DAPI (blue). **(F)** Quantification of cell cross-sectional area by measuring 100 random cells. **(G)** HW/BW and heart weight-to-tibia length ratios in each group. **(H)** The mRNA expression of hypertrophy-associated genes. β-actin was used as the internal control. ANP, atrial natriuretic peptide. BNP, brain natriuretic peptide. β-MHC, β-myosin heavy chain. ^###^*P* < 0.001 compared with sham, ^##^*P* < 0.01 compared with sham; ***P < 0.001 compared with TAC, **P < 0.01 compared with TAC, *P < 0.05 compared with TAC.

Next, we determined whether SMYAD administration attenuated cardiac hypertrophy and HF. The samples shown in **Figure 2A** and HE staining of whole heart (**Figure 2B**) demonstrated that TAC mice have dilated hearts and SMYAD administration prevented left ventricle dilation. Histological analysis indicated that TAC caused significant increase of cardiac myocyte cross-sectional area. WGA staining of histological sections further confirmed the inhibitory effect of SMYAD on cardiac hypertrophy induced by TAC (**Figures 2E, F**). As shown in **Figure 2G**, pressure overload induced by TAC remarkably increased the ratio of heart weight to body weight (HW/BW) and the ratio of heart weight to tibia length compared by the sham group (*P* < 0.001). In contrast, SMYAD administration effectively reduced the ratio of HW/BW by 22.2% compared with the TAC group (*P* < 0.01). Consistently, real-time polymerase chain reaction analysis of cardiac hypertrophy markers, such as atrial natriuretic peptide, brain natriuretic peptide, and β-myosin heavy chain, manifested a significant alleviation in the presence of SMYAD compared with that in TAC-treated mice (**Figure 2H**). Together, these data indicated that SMYAD prevented the development of cardiac hypertrophy *in vivo*.

### Si-Miao-Yong-An Decoction Attenuated Histological Changes Following Transverse Aortic Constriction Surgery

According to HE staining, the heart tissues of the sham group displayed normal myofibrils with neatly arrangement. In the TAC group, a dense inflammatory infiltration, necrosis of a large number of cardiomyocytes, muscle fiber dissolution, and the disappearance of the normal structure of the myofibrils were observed. Heart tissues in the SMYAD and captopril groups showed less necrosis of myocardial cell than that in the TAC group. In addition, we found that necrosis of cardiomyocytes were induced both interstitial and perivascular in TAC hearts and attenuated in SMYAD-treated hearts (**Figures 2C, D**).

### Si-Miao-Yong-An Decoction Inhibited CD41- and CD61-Mediated Platelet Aggregation in TAC-Induced Heart Failure

**Figure 3:**
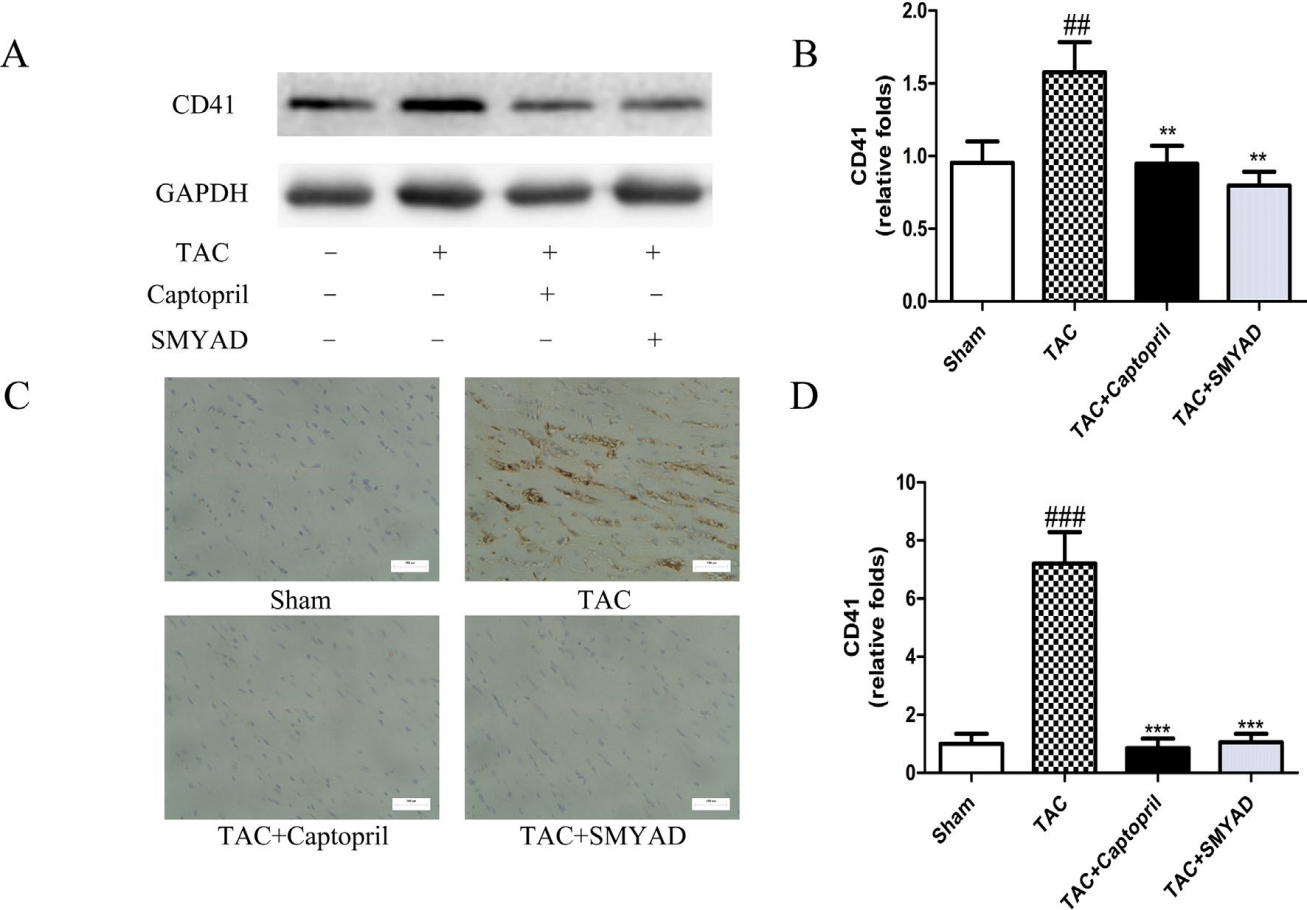
SMYAD suppressed CD41 expression in mice with TAC. **(A** and **B)** Protein level of CD41 in sham, TAC, TAC + captopril, and TAC + SMYAD was verified by western blot. **(C** and **D)** Expression of CD41 in each group was verified by immunohistochemistry examination. ^###^*P* < 0.001, ^##^*P* < 0.01 vs the sham group; ***P < 0.001, **P < 0.01 vs the TAC group.

**Figure 4:**
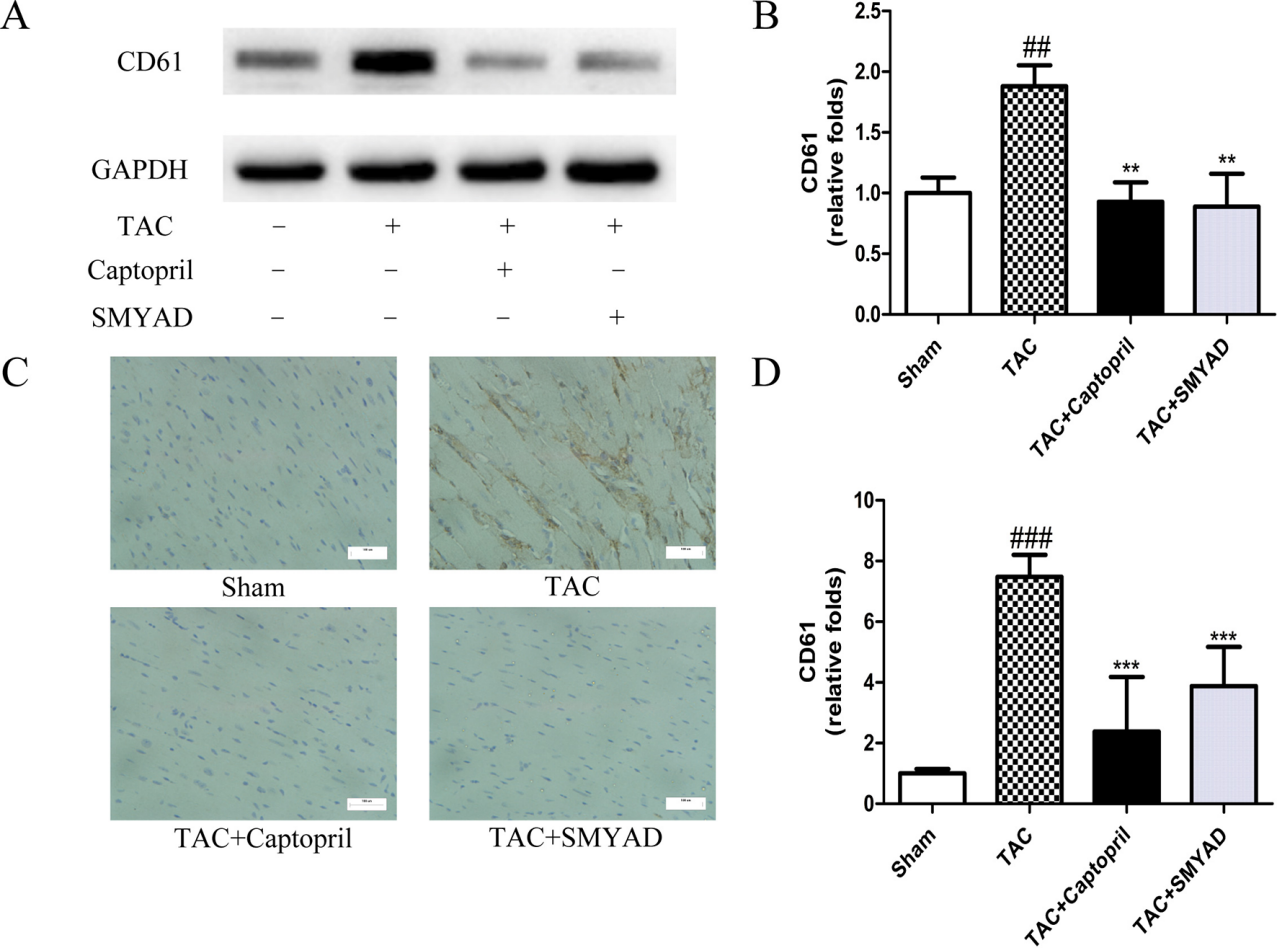
SMYAD suppressed CD61 expression in mice with TAC. **(A** and **B)** Expression of CD61 protein in each group was verified by Western blot examination. **(C** and **D)** Representative immunostaining and quantification of CD61 by immunohistochemistry examination at 4 weeks post-surgery. ^###^*P* < 0.001, ^##^*P* < 0.01 vs the sham group; ***P < 0.001, **P < 0.01 vs the TAC group.

To confirm that platelet aggregation is altered in hearts after TAC surgery, we examined CD41 and CD61 levels in hearts at 4 weeks after TAC surgery. **Figures 3A, B** and **4A, B** exhibited that CD41 and CD61 expressions were increased in TAC mice compared with those in the control mice. However, both CD41 and CD61 were significantly reduced in the SMYAD-treated mice. Furthermore, immunohistochemistry staining of heart tissues illustrated that TAC treatment significantly increased the accumulation of CD41 and CD61 (*P* < 0.001) (**Figures 3C, D** and **4C, D**). Compared with those in the TAC group, CD41 and CD61 expressions were significantly decreased in the TAC + SMYAD group (**Figures 3C** and **4C**).

### Si-Miao-Yong-An Decoction Reduced Platelet Activation in Heart Tissue

**Figure 5:**
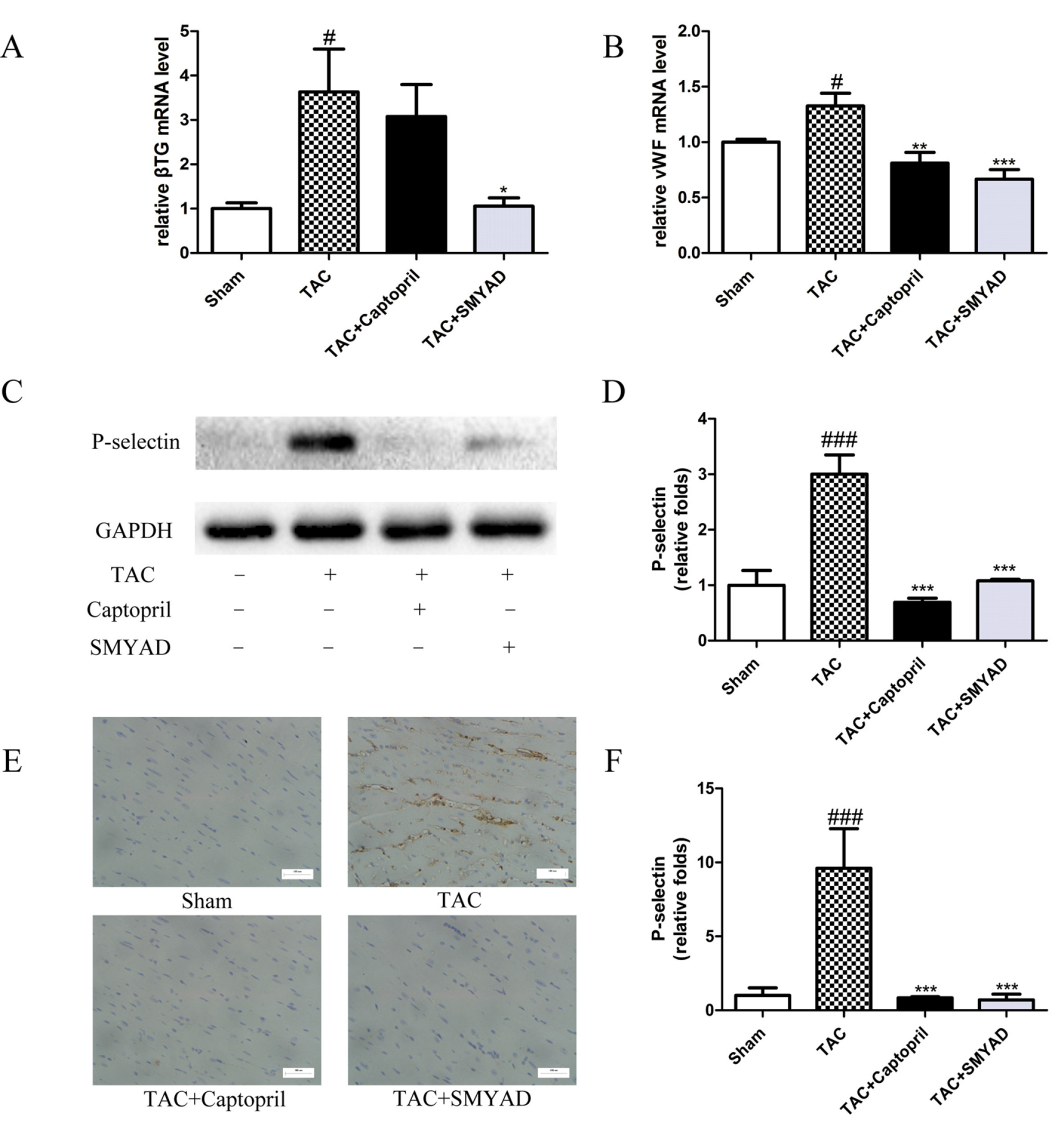
SMYAD suppressed platelet activation in mice with TAC. **(A** and **B)** mRNA level of β-TG and vWF in each group. β-TG, β-thromboglobulin. vWF, von Willebrand factor. **(C** and **D)** Representative immunoblots and calculation of P-selectin levels in the sham, TAC, TAC + captopril, and TAC+ SMYAD. **(E** and **F)** Expression of P-selectin protein in the myocardium was detected by histopathological staining. ^###^*P* < 0.001 compared with sham, ^#^*P* < 0.05 compared with sham; ***P < 0.001 compared with TAC, **P < 0.01 compared with TAC, *P < 0.05 compared with TAC.

β-thromboglobulin (β-TG) is a specific protein that secreted from the α-granules of platelet during the release reaction. von Willebrand factor (vWF) is critical for normal platelet tethering during hemostasis. vWF unfolds from its inactive globular conformation into an active string-like form that can specifically recruit platelets in response to blood shear forces. In our study, β-TG and vWF messenger RNA (mRNA) expression levels were increased by 3-fold and 1.3-fold, respectively, in TAC mice compared with those in sham, which were both reduced in SMYAD group (**Figures 5A, B**). P-selectin is a marker of activated platelet following heart injury. Thus, we investigated the possible involvement of P-selectin in the cardioprotective activity of SMYAD against cardiac hypertrophy by immunohistochemical staining and Western blot. In accordance to previous reports, TAC treatment significantly increased the expression of P-selectin protein as compared with sham group. SMYAD significantly attenuated the upregulation of P-selectin protein in TAC-treated mice (**Figures 5C, D**). Meanwhile, we observed that the levels of P-selectin in the TAC group were significantly increased compared with those in the sham-operated group based on immunohistochemical staining (**Figures 5E, F**). SMYAD treatment significantly suppressed the expression of P-selectin compared with the TAC group. In conclusion, our results suggested that SMYAD mediated the inhibitory effects on cardiac hypertrophy by disrupting both platelet aggregation and platelet activation.

## Discussion

HF represents a heterogeneous condition characterized by vulnerabilities in the blood, vasculature, and impaired flow dynamics that predispose to both arterial and venous thrombosis. Despite the fact that treatment with angiotensin-converting enzyme inhibitors, angiotensin receptor blockers, beta adrenergic antagonists, and mineralocorticoid receptor antagonists in HF patients has improved long-term outcomes, morbidity or mortality remain high in patients with acute decompensated HF (Zannad et al., 2013). Furthermore, there has been little progress in preventing the adverse cardiac remodeling that initiates HF.

Immune activation, inflammation, oxidative stress, alterations in mitochondrial bioenergetics, and autophagy have been postulated as important pathophysiological events in HF (Ayoub et al., 2017). It is commonly recognized that platelet activation plays a main role in cardiovascular disease. There is evidence that platelet activation and hypercoagulability present in HF, and stroke were reduced by warfarin therapy in the HF patient (Gurbel and Tantry, 2014). Platelets normally maintained a quiescent state and were sensitive to homeostasis changes resulting from endothelial cell damage, stress conditions, and hemodynamic abnormality (Hansen et al., 2015). A variety of adhesion molecules were prestored inside platelet granules and expressed on the platelet surface responding to activation. β-TG and platelet factor 4 represented specific platelet proteins of alpha-granules, which could be secreted into surrounding medium during cell activation. It was demonstrated that β-TG and P-selectin were significantly associated with the occurrence of atrial fibrillation and consequent stroke (Weymann et al., 2017). Thus, platelet activation contributes to the progress of cardiovascular disease.

SMYAD is a famous traditional Chinese medicine formula that was mainly applied to treat thromboangiitis obliterans in China for centuries and presented satisfactory curative effect. The main function of SMYAD is activation of blood circulation to dissipate blood stasis and promote vital energy regulation. Clinically, SMYAD was reported to improve myocardial infarction and HF (Zhou et al., 2012). Recently, SMYAD has been verified to improve liver function and reduce fat accumulation in hyperlipidemia rat by accelerated transformation of cholesterol into bile acids (Liu et al., 2017b). It has not been reported whether SMYAD had effect on cardiac dysfunction induced by TAC. Thus, our study is aimed to investigate the effects and mechanisms of SMYAD on cardiac hypertrophy.

We demonstrated that SMYAD improved systolic function in HF mice, as well as modestly reduced LVID and LV Vol. However, the molecular mechanism by which SMYAD mediates its anti-hypertrophic effects remains unclear, and the signaling pathways that interact to drive hypertrophy are very complicated. To understand the molecular mechanisms responsible for SMYAD’s effects in HF, we investigated platelet, which has been shown to be activated in TAC-treated mice to induce cardiomyocyte hypertrophy and collagen synthesis (Wu et al., 2017a). We observed increased activation of cardiac CD41/61 and P-selectin in TAC-treated mice, as well as the reverse effect of SMYAD. P-selectin (CD62P) is a member of the selectin family of cell adhesion molecules and is stored on the membrane of platelet α-granules and endothelial Weibel–Palade bodies (Qi et al., 2015). It was the main platelet activation marker appearing on the surface of platelet membrane (Xie et al., 2014). It was reported that the expression of platelet P-selectin was increased in hyperlipidemic patients after ischemic stroke (Pawelczyk et al., 2015). In our study, SMYAD reduced the activation of platelet by downregulating P-selectin. P-selectin is responsible for the adhesion of certain leukocytes and platelets to the endothelium when thrombosis develops and inflammation is caused. It rapidly translocated to the cell surfaces of platelets and endothelial cells upon activation (Qi et al., 2015). Platelets can induce cell survival and growth by secreting several sorts of growth factors, including vascular endothelial growth factors, fibroblast growth factors, and platelet-derived growth factors. The plasma concentration of soluble P-selectin is now recognized as a predictor of adverse cardiovascular events (Ishida et al., 2014).

CD41 and CD61 are the main receptors mediating platelet aggregation, and CD61 is the most abundant receptor expressed on the platelet surface. Increased expression of CD61 on platelet surfaces results in enhanced fibrinogen binding and, subsequently, platelets cross-linking and thrombogenesis. SMYAD is suggested to inhibit platelet aggregation by CD41/61 expression and the elevation of vWF. CD41/61 and P-selectin significantly increased in atrial fibrillation patient with hypercoagulable state (Zhang et al., 2018). Besides, the prothrombotic state was characterized by the expression of CD41/61 and P-selectin mediating binding of vWF and fibrin interactions that led to different abnormalities in platelet function (Karmakar et al., 2015). In our study, inhibition of platelet activation by SMYAD reduced the infiltration of inflammatory cell into the heart.

Anticoagulants (e.g., warfarin) and antiplatelet agents (e.g. aspirin) are the principle antithrombotic agents. Many HF patients with sinus rhythm took aspirin because coronary artery disease was the leading cause of HF (Shantsila and Lip, 2016). The prescription of antiplatelet agents, such as clopidogrel and aspirin, has indeed become a stronghold of atherosclerotic cardiovascular disease and heart attack therapy. The therapeutic efficiency of clopidogrel is manipulated by the actions of hepatic cytochrome P450 (CYP) enzymes and influenced by individual genetic variations. The metabolism of clopidogrel into its active metabolites is affected by individual polymorphisms of CYP enzymes (Jarrar et al., 2016). Patients with high on-treatment platelet reactivity upon clopidogrel were at increased risk for thrombotic events, particularly for stent thrombosis and myocardial infarction, but cardiovascular mortality was also elevated in patients undergoing percutaneous coronary intervention (Gross et al., 2016). It was reported that aspirin use after discharge for HF hospitalization was associated with reduced risk of death, all-cause readmission, and HF readmission (Kim et al., 2016). However, data on aspirin in HF were inconclusive because of bleeding complications. There was evidence that a lower risk for death or stroke was associated with warfarin therapy in the HF patient (Hart et al., 2007). Warfarin (vitamin K antagonist) blocks multiple steps of coagulation by reducing the synthesis of vitamin K-dependent coagulation factors. As warfarin was metabolized by the CYP isoenzymes, the pharmacokinetics and pharmacodynamics of warfarin were influenced by interactions with other drugs that were metabolized by the same CYP isoenzymes (Gurbel and Tantry, 2014). Thus, it is of great urgency to find anti-platelet drugs with fewer adverse reaction.

Persistent hemodynamic overload will result in pathological cardiac hypertrophy, which is a leading predictor for the development of HF. In the present study, we used a TAC mice model to mimic pressure overload-induced stress. We demonstrated that SMYAD improved pressure overload-induced cardiac dysfunction and remodeling, and promoted histological changes in heart tissues. We also found that SMYAD decreased protein expressions of platelet aggregation markers (CD41, CD61) and platelet activation marker (P-selectin) in pressure overload-induced failing hearts. We have previously isolated and identified some chemical constituents, but the relationship between pharmacological effects and chemical constituents needs to be further studied.

The healthy endothelium in large vessels has multiple mechanisms that inhibit the adhesion or activation of platelets, either directly or by actively degrading platelet agonists. One of the earliest events in atherosclerosis is the loss of normal endothelial functions, including disruption of anti-platelet mechanisms. Endothelial cells respond to injury by releasing numerous factors, including vWF. vWF is an established marker of endothelial activation, and patients with elevated plasma levels of the ultra large vWF molecules are at high risk for future cardiovascular events (Warlo et al., 2017). vWF is a glycoprotein involved in both platelet activation and aggregation through its binding sites for GpIb and GpIIb/IIIa, respectively. High circulating levels of unusually large vWF multimers have strong procoagulant activity and facilitate platelet adhesion and aggregation by interacting with platelets after an acute event superimposed on coronary artery disease (Akyol et al., 2016). There was increasing evidence that platelet–endothelial interactions also contributed to early atherosclerotic plaque initiation and growth (Wu et al., 2017b). Platelets may also play a key role in neutrophil extracellular traps (Carestia et al., 2016).Through these interactions, platelet-derived factors can contribute to the pro-inflammatory and mitogenic status of resident mural cells. There was evidence that transendothelial migration of platelet monocyte complexes may result in dissociation and surface deposition of platelets (van Gils et al., 2008). In the next study, we will further investigate the interaction between platelet, endothelial cells, and neutrophil.

## Conclusions

In this study, we characterized the property of SMYAD against cardiac hypertrophy. SMYAD inhibited pressure overload-induced platelet activation, cardiac hypertrophy, and dysfunction. It inhibited TAC-induced hypertrophy, myocardial necrosis, platelet aggregation, and platelet activation. CD41/CD61 served as a mediator of the platelet aggregation effect, while P-selectin/β-TG mediated the platelet activation effect. This study provided evidence that SMYAD may be a promising therapeutic agent for adverse cardiac remodeling.

## Data Availability

All datasets generated for this study are included in the manuscript and/or the **Supplementary files**.

## Ethics Statement

All the animal experiments were performed in accordance with the “Guide for the Care and Use of Laboratory Animals” by the National Institutes of Health. All studies were conducted in line with the approval of the Animal Care Committee of Beijing University of Chinese Medicine.

## Author Contributions

CS, SG, WW, and SL conceived and designed the experiments. CS, QW, HZ, WJ, and HL carried out the experiments, analyzed the data, and drafted the manuscript. BL, WW, XY, LL, and XC participated in its design and prepared the paper. SL, SG, and QW revised the manuscript. All authors read and approved the final manuscript.

## Funding

This study was funded by the National Nature Science Foundation of China (grant NO. 81874387, 81774101, and 81703942) and the Fundamental Research Funds for the Central Universities (2019-JYB-TD-002 and 2019-JYB-XS-012).

## Conflict of Interest Statement

The authors declare that the research was conducted in the absence of any commercial or financial relationships that could be construed as a potential conflict of interest.
