# Supplementary material for: Si-Miao-Yong-An Decoction Protects Against Cardiac Hypertrophy and Dysfunction by Inhibiting Platelet Aggregation and Activation
Source: Front Pharmacol. 2019 Sep 18;10:990. doi: 10.3389/fphar.2019.00990 (PMC6759602; doi:10.3389/fphar.2019.00990)
Supplement: Supplementary file 1 [file Table_1.docx]

**Supplementary Table 1|** Detailed information of the crude drugs composed in SMYAD.

| **No.** | **Drug Name** | **Authentication** | **Voucher specimen** |
| --- | --- | --- | --- |
| SMYAD-A | Lonicerae Japonicae Flos | Flower bud of *Lonicera japonica Thunb.* | 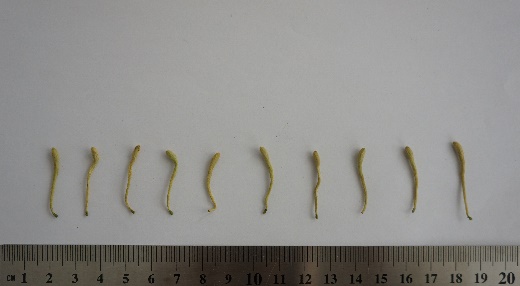 |
| SMYAD-B | Scrophulariae Radix | Root of *Scrophularia ningpoensis Hemsl* | 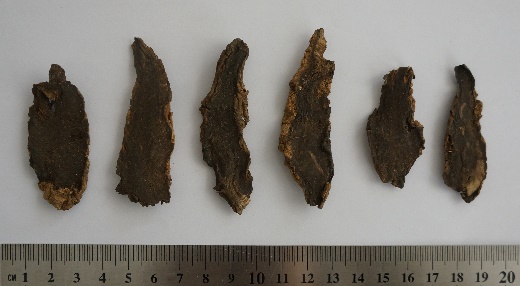 |
| SMYAD-C | Angelicae Sinensis Radix | Root of. *Angelica sinensis* ( Oliv. ) Diels | 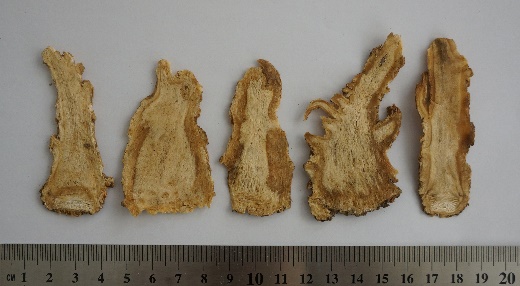 |
| SMYAD-D | Glycyrrhizae Radix Et Rhizoma | Stem of *Glycyrrhiza uralensis* Fisch | 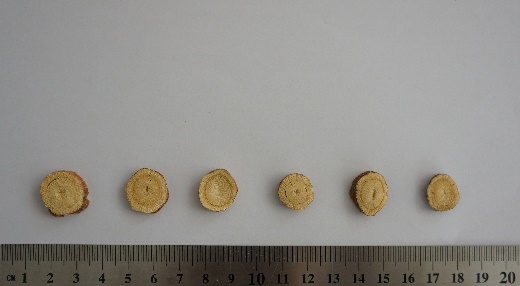 |
